# Soft Drinks and Symptoms of Depression and Anxiety in Overweight Subjects: A Longitudinal Analysis of an European Cohort

**DOI:** 10.3390/nu15183865

**Published:** 2023-09-05

**Authors:** Adoración Castro, Margalida Gili, Marjolein Visser, Brenda W. J. H. Penninx, Ingeborg A. Brouwer, Juan José Montaño, María Ángeles Pérez-Ara, Mauro García-Toro, Ed Watkins, Matt Owens, Ulrich Hegerl, Elisabeth Kohls, Mariska Bot, Miquel Roca

**Affiliations:** 1 Research Institute of Health Sciences (IUNICS), University of the Balearic Islands (UIB), 07122 Palma de Mallorca, Spain; mgili@uib.es (M.G.); marian@aliaspsicologia.com (M.Á.P.-A.); mauro.garcia@uib.es (M.G.-T.); mroca@uib.es (M.R.); 2 Health Research Institute of the Balearic Islands (IdISBa), Hospital Universitario Son Espases, Edificio S, 07120 Palma de Mallorca, Spain; juanjo.montano@uib.es; 3 Department of Psychology, University of the Balearic Islands (UIB), 07122 Palma de Mallorca, Spain; 4 Department of Health Sciences, Faculty of Science, Amsterdam Public Health Research Institute, Vrije Universiteit Amsterdam, 1081 HV Amsterdam, The Netherlands; m.visser@vu.nl (M.V.); ingeborg.brouwer@vu.nl (I.A.B.); 5 Department of Psychiatry, Amsterdam Public Health Research Institute, Amsterdam UMC, Vrije Universiteit Amsterdam, De Boelelaan 1117, 1081 HV Amsterdam, The Netherlands; b.penninx@amsterdamumc.nl (B.W.J.H.P.); m.bot1@amsterdamumc.nl (M.B.); 6 Department of Medicine, University of the Balearic Islands (UIB), 07122 Palma de Mallorca, Spain; 7 Department of Psychology, University of Exeter, Exeter EX4 4PY, UK; e.r.watkins@exeter.ac.uk (E.W.); m.owens-solari@exeter.ac.uk (M.O.); 8 Department of Psychiatry, Psychosomatics and Psychotherapy, Goethe Universität Frankfurt am Main, 60528 Frankfurt am Main, Germany; ulrich.hegerl@deutsche-depressionshilfe.de; 9 Department of Psychiatry and Psychotherapy, Medical Faculty, University Leipzig, 04109 Leipzig, Germany; elisabeth.kohls@medizin.uni-leipzig.de

**Keywords:** depression, anxiety, soft drinks, coffee, tea

## Abstract

Background: Studies about the association of carbonated/soft drinks, coffee, and tea with depression and anxiety are scarce and inconclusive and little is known about this association in European adults. Our aim was to examine the association between the consumption of these beverages and depressive and anxiety symptom severity. Methods: A total of 941 European overweight adults (mean age, 46.8 years) with subsyndromal depression that participated in the MooDFOOD depression prevention randomized controlled trial (Clinical Trials.gov identifier: NCT2529423; date of the study: from 2014 to 2018) were analyzed. Depressive and anxiety symptom severity and beverage consumption were assessed using multilevel mixed-effects ordinal logistic regression models for each beverage consumption (carbonated/soft drink with sugar, carbonated/soft drink with non-nutritive sweeteners, coffee, and tea) with the three repeated measures of follow-up (baseline and 6 and 12 months). A case report form for participants’ sociodemographic and clinical characteristics, the Food Frequency Questionnaire, the Patient Health Questionnaire-9, the Generalized Anxiety Disorder 7-Item Scale, the MINI International Neuropsychiatric Interview 5.0, the Short Questionnaire to Assess Health-Enhancing Psychical Activity, and the Alcohol Use Disorders Identification Test were the research tools used. Results: Daily consumption of carbonated/soft drinks with sugar was associated with a higher level of anxiety. Trends towards significance were found for associations between both daily consumption of carbonated/soft drinks with sugar and non-nutritive sweeteners and a higher level of depression. No relationship was found between coffee and tea consumption and the level of depression and anxiety. Conclusions: The high and regular consumption of carbonated/soft drink with sugar (amount of consumption: ≥1 unit (200 mL)/day) tended to be associated with higher level of anxiety in a multicountry sample of overweight subjects with subsyndromal depressive symptoms. It is important to point out that further research in this area is essential to provide valuable information about the intake patterns of non-alcoholic beverages and their relationship with affective disorders in the European adult population.

## 1. Introduction

According to the World Health Organization (WHO), depression and anxiety are the two most common mental disorders. Worldwide, the total number of people living with depression is 322 million and living with anxiety is 264 million, being 40.27 and 36.17 million, respectively, in the European area [1]. Both disorders are associated with substantial losses in health and functioning [1]. Mental disorders are responsible for 13% of the total burden of disease, similar to other illnesses such as cardiovascular and circulatory diseases [2].

It has been proposed that different dietary habits, foods, beverages, and nutrients may impact on the appearance, course, or severity of psychological disorders [3]. Coffee, tea, and soft drinks are the most consumed beverages without alcohol worldwide [4].

In recent decades, high intake of soft drinks has emerged as a significant public health problem [5,6]. Previous research showed that high consumption of sugar-sweetened soft drinks was related to a higher prevalence of a history of major depressive disorder (MDD) [7] and to a higher prevalence of symptoms of depression in adolescents and adults [8,9,10,11,12]. Evidence about the association between soft drinks consumption and anxiety symptomatology is mostly focused on adolescents: students who consumed soft drinks ≥7 times per week had significantly higher anxiety symptomatology compared with non-consumers [9].

Coffee and tea are the world’s major sources of dietary caffeine [13]. The literature shows an inverse association between coffee consumption and depressive symptoms [14,15,16]. 

The association between tea and depressive symptomatology is not free from a certain geographical bias (most of the studies are from Asian populations) and the results found are divergent. In a large cross-sectional and prospective study, no association was found between the consumption of green tea and depressive symptoms in a working population in Japan [17]. Regarding anxiety symptomatology, a recent systematic review indicates that a supplementation of L-theatine (L-THE) may help in the reduction of stress and anxiety in those who are experiencing stressful situations [18]. Similar results were found in another systematic review, which included observational studies and RCTs: consumption of green tea influenced the reduction in anxiety symptoms [19].

Concerning caffeine, its consumption can attenuate depressive symptomatology, whereas high doses of caffeine may induce anxiety [16,20,21]. In a large cross-sectional study of adults from the US, after controlling for potential confounders, an inverse link between caffeine consumption and depressive symptomatology was found, suggesting that its psychostimulant properties appear to protect against depressive symptoms [22].

In a previous work, we explored the relationship between these non-alcoholic beverages and MDD, as well as the differences in mood, cognitive, and somatic/vegetative depression clusters [7]. However, studies about the association of these beverages with the severity of anxiety and depression are scarce and inconclusive, and little research has been conducted in European countries. To address other possible relationships between depression and anxiety symptomatology and beverage consumption, the aim of the present study was to examine the association between the consumption of carbonated/soft drinks, coffee, and/or tea with depressive and anxiety symptom severity using data from a large-scale European prevention trial (the MooDFOOD trial) [23].

## 2. Materials and Methods

### 2.1. Study Design

This study is a secondary analysis of the MooDFOOD study (Clinical Trials.gov identifier: NCT2529423), whose objective was to assess the feasibility and effectiveness of nutritional strategies to prevent an episode of MDD in overweight people with subsyndromal depressive symptomatology. Participants were randomized into one of the following intervention groups: (1) daily multi-nutrient supplements; (2) placebo; (3) daily multi-nutrients supplements plus food-related behavioral activation therapy (F-BA); and (4) placebo plus F-BA. The research protocol and main results have been reported elsewhere [23,24].

### 2.2. Intervention Groups Description

#### 2.2.1. Multi-Nutrient Supplements

Participants received either multi-nutrient supplements or placebo, administered in 2 capsules per day, taken daily for 1 year. Multi-nutrient supplements were specifically prepared for the study and consisted of one pill containing omega-3 fatty acids, 1412 mg of eicosapentaenoic acid and docosahexaenoic acid, and a pill containing selenium, folic acid, and vitamin D3 coupled with calcium. The placebo was identical in shape, color, and packaging to the multi-nutrient supplement but contained sunflower oil (pill 1) and filler materials (pill 2).

#### 2.2.2. Food-Related Behavioral Activation Therapy (F-BA)

The F-BA consisted of different nutritional advice on improving food-related behaviors and making dietary shifts into a healthy Mediterranean-style diet. This MooDFOOD diet promoted specific intake amounts for ten different food groups. The intervention comprised a maximum of 21 individual and group-based sessions for one year, delivered by psychologists familiar with BA.

Full details of the interventions and main results have been reported elsewhere [23,24] and the findings showed that multi-nutrient supplements compared with placebo and FB-A and with no therapy did not affect MDD onset in overweight subjects with subsyndromal depressive symptoms.

### 2.3. Participants

In the original investigation, a total of 1025 participants were recruited and included between September 2015 and October 2016 in the Netherlands, United Kingdom, Germany, and Spain. Participants were included if they were aged between 18 and 75 years old; met the criteria for overweight or obesity (body mass index (BMI) between 25 and 40 kg/m^2^); and reported subsyndromal symptoms of depression (score of at least 5 points assessed by the Patient Health Questionnaire (PHQ-9)) [25]. We excluded participants who were in a current episode of MDD according to the psychiatric Diagnostic and Statistical Manual of Mental Disorders, Fourth Edition (DSM-IV) [26] criteria (in the past 6 months) evaluated by the structured MINI International Neuropsychiatric Interview 5.0 (MINI 5.0) [27]; had a current eating disorder; had a history of psychosis, bipolar disorder, substance dependence, or other severe psychiatric disorder that requires specialized clinical attention; history of bariatric surgery; currently pregnant or breastfeeding; current severe life-threatening physical disease; had a severe cognitive impairment limiting the participation in the study, assessed by the ability to complete the screening instruments in an adequate manner; currently receiving a behavioral intervention that interferes with MooDFOOD prevention trial intervention; and finally, unwilling to stop using specific dietary supplements that are used or are competing with the MooDFOOD prevention trial.

For this study, a total of 941 participants were analyzed (those who completed the Food Frequency Questionnaire (FFQ) in each study assessment). Those with no or incomplete data on the FFQ were excluded (*n* = 84).

### 2.4. Procedure

Different strategies were used to recruit participants, mainly by presenting the study in public areas, internet, social media, newspapers, as well as in general practice settings and other registers, to make the information easily available at the four sites.

Inclusion and exclusion criteria were checked with a brief screening questionnaire, follow-up by a telephonic interview. A face-to-face baseline assessment, conducted by experienced researchers, was conducted after inclusion and informed consent. Baseline measurements included interviews for the assessment of a lifetime history of depression, through the MINI 5.0 depression section [27], sociodemographic data, physical measurements, and self-administered questionnaires. They were assigned to one of four interventions described above. Measurements took place at baseline, and at the 3-, 6-, and 12-month follow-up and after 24 months (online only). Nevertheless, for this analysis, only 0, 6, and 12 months were used. All participants provided their informed consent for inclusion before being included in the MooDFOOD study.

### 2.5. Instruments

#### 2.5.1. Beverages Consumption Variables

Carbonated/soft drinks, coffee, and tea consumption were collected through the Food Frequency Questionnaire (GA2LEN-FFQ) [28] at baseline, and 6- and 12-month follow-up. The FFQ includes an extensive range of foods and nutrients according to the European diet. For this analysis, the “non-alcoholic beverages” section was used: carbonated/soft drinks with sugar or with non-nutritive sweeteners (NNSs) assessed in units of 200 mL and tea/coffee assessed in units of “a cup”. Consumption frequency was evaluated in the following categories: never/rarely, 1–3 times a month, once a week, 2–4 times a week, 5–6 times a week, once a day, 2–3 times a day, and 4 or more times a day. For the purposes of this study, consumption frequency was categorized as follows: <1 unit a week, 1 to 6 units a week, 1 or more units a day for carbonated/soft drinks and <1 cup a day, 1 to 3 cups a day, >3 cups a day for coffee and tea.

#### 2.5.2. Primary Outcomes

Primary outcomes were depression severity, assessed by the Patient Health Questionnaire-9 (PHQ-9) [25], range 0–27, with higher scores indicating higher severity and anxiety severity, and the Generalized Anxiety Disorder 7-Item Scale (GAD-7), range 0–21, with higher scores indicating higher severity [29]. Both were measured at baseline, and 3-, 6-, and 12-month follow-up.

#### 2.5.3. Covariates

Participants’ sociodemographic characteristics (age, sex, site, and educational level) were gathered at baseline. The presence of lifetime MDD were evaluated at baseline, and 3-, 6-, and 12-month follow-up through the MINI International Neuropsychiatric Interview 5.0 (MINI 5.0) [27]. The Adherence to the MooDFOOD diet, estimated in a range from 0 to 77, with higher values indicating a better adherence, based on the GA2LEN-FFQ [28]. Physical activity was evaluated at baseline, and 3-, 6-, and 12-month follow-up through the last item from the Short Questionnaire to Assess Health-Enhancing Psychical Activity (SQUASH) [30]. Risk of alcohol consumption, dependence symptoms, and harmful alcohol consumption was measured at baseline, and 3-, 6-, and 12-month follow-up by the Alcohol Use Disorders Identification Test (AUDIT), range of 0–40, indicating the level of risk and dependence of alcohol consumption [31]. Height and weight were asked at baseline, and 3-, 6- and 12-month follow-up to calculate the BMI. The presence of diabetes was asked at the baseline interview.

### 2.6. Data Analyses

For descriptive analyses, mean and standard deviation (SD) for continuous data and frequency (and percentage) for categorical data were calculated.

The Shapiro–Wilk test for normality indicated that the PHQ-9 and GAD-7 scores did not follow a normal distribution at any recorded time point (*p* < 0.01). The application of Box–Cox and Johnson transformations failed to convert these variables into normal distributions. For this reason, both variables were grouped into categories. For the PHQ-9, the following categories were used: 0—Minimal (scores 0–4), 1—Mild (scores 5–9), 2—Moderate (scores 10–14), and 3—Moderate-Severe (scores 15–27). For the GAD-7, the following categories were used: 0—Minimal (scores 0–4), 1—Mild (scores 5–9), and 2—Moderate-Severe (scores 10–21).

Multilevel mixed-effects ordered logistic regression models were constructed for consumption of each beverage (carbonated/soft drink with sugar, carbonated/soft drink with NNSs, coffee, and tea) with the three repeated measures (baseline, 6 and 12 months) on depressive and anxiety symptomatology severity groups as outcome variables. In these models, the random effects refer to the subject level. Marginal effects analysis allowed us to study the association between each level of consumption and each level of depression and anxiety. To fit the association of the consumption of different beverages to depression and anxiety symptoms, a set of covariates were included. The following continuous variables were age, alcohol use, physical activity, BMI, and MooDFOOD diet score. Categorical variables were coded as dummy variables: sex (male vs. female); site (Germany vs. Spain vs. the United Kingdom vs. The Netherlands); level of education (intermediate vs. high vs. low); group intervention (Placebo plus F-BA vs. Supplements without FB-A vs. Supplements plus FB-A vs. Placebo without FB-A); history of MDD diagnosis (yes vs. no); and diabetes (yes vs. no). Beverage consumption was coded as time-dependent variables at all three measurements (baseline, 6 and 12 months): soft drink consumption was classified as <1 unit a week, 1 to 6 units a week, and 1 or more units a day; coffee and tea consumption was classified as <1 cup a day, 1 to 3 cups a day, and >3 cups a day. Although the FFQ scale is composed of 8 categories of consumption, we grouped it into these 3 categories for statistical reasons. Finally, analyses were performed using STATA 16 [32]. *p* values < 0.05 were considered significant. 

## 3. Results

### 3.1. Sample Description

Final analysis was undertaken using data from 941 participants who completed the FFQ at baseline and follow-up assessments, with 24.9% receiving Placebo without FB-A, 25.7% receiving Placebo plus FB-A, 24.3% receiving supplements without FB-A, and 25.1% receiving supplements plus FB-A. The sample was predominantly composed of women (75.3%) and the overall mean age was 46.8 (SD = 12.9) years. The BMI mean was 31.3 (SD = 3.95) kg/m^2^, the mean score of the AUDIT test was 3.88 (SD = 3.67), the participants performed physical activity on average 3.61 (SD = 2.34) days/week, and 5.2% had diabetes. Regarding depressive symptomatology, the PHQ-9 mean was 7.43 (SD = 4.23) and almost half of the sample (48%) was in the mild symptomatology category. In terms of anxiety severity, the GAD-7 mean was 5.76 (SD = 4.11) and 45.7% of the participants was in the minimal symptomatology category. Concerning beverage consumption, 77.7% of the participants drank less than one glass per week of carbonated/soft drinks with sugar, 64% drank less than one glass per week of carbonated/soft drink with NNSs, 56,7% drank 1–3 cups per day of coffee and 66.6% drank less than a cup of tea per day. The sample group’s characteristics are shown in Table 1.

### 3.2. Multilevel Mixed-Effects Ordinal Logistic Regression Analyses Results

Table 2 shows associations between beverage consumption and depression and anxiety (at baseline, 6- and 12-month follow-up) symptomatology severity categories in multilevel mixed-effects ordered logistic regression adjusted models.

The eight models relating the consumption of each type of beverage and the level of depression and the level of anxiety were adjusted by time, age, sex, site, level of education, BMI, MooDFOOD diet score, alcohol use, physical activity, diabetes, history of major depressive disorder diagnosis, and group intervention.

The results show that daily consumption of carbonated/soft drinks with sugar was associated with a higher level of anxiety (OR = 2.51; *p* = 0.033, 95%IC(OR): 1.075, 5.864). Marginal effects analysis shows an association between daily consumption of this beverage and mild anxiety (*p* = 0.008). A trend towards significance can also be observed in the association between daily consumption of carbonated/soft drinks with sugar and a higher level of depression (OR = 1.965, *p* = 0.062, 95%IC(OR): 0.967, 3993). In this sense, marginal effects analysis shows an association between daily consumption of this beverage and mild depression (*p* = 0.003). Finally, a trend towards significance can also be observed in the association between daily consumption of carbonated/soft drinks with NNSs and a higher level of depression (OR = 1.57, *p* = 0.075, 95%IC(OR): 0.955, 2.581). Marginal effects analysis shows an association between daily consumption of this beverage and mild depression (*p* = 0.03).

Finally, coffee and tea consumption were not shown to be associated with depression and anxiety levels in any of the amounts of consumption analyzed.

## 4. Discussion

This study examined the association between the consumption of carbonated/soft drinks, coffee, and tea and depressive and anxiety symptom severity using data from the European depression prevention trial MooDFOOD. The results show that carbonated/soft drinks were associated with a higher level of anxiety. Specifically, people who drink more than one unit (200 mL) per day of carbonated/soft drinks with sugar have greater symptoms of anxiety compared to those who drink less than one unit per week.

These findings are in line with findings from adolescents [9]. Among the adult population, the evidence is scarce, and the results are contradictory. On the one hand, a study carried out among Australian adults showed that high soft drink consumption was associated with several mental health problems, such as depression, suicidal ideation, psychological distress, and a current mental health condition, but not anxiety [33]. On the other hand, Boaz et al. [34] examined the relationship between diet and psychological stress during the COVID-19 pandemic in 3271 participants around the world. Anxiety level, according to the GAD-7, was significantly and positively linked with the number of sweetened beverages consumed per day. However, this research was carried out during the initial phase of COVID-19, and it is well established that this pandemic had an adverse effect on healthy lifestyles, decreasing mental health and quality of life [35].

Also, our results show a trend towards significance in the association between both daily consumption of carbonated/soft drinks with sugar and with NNSs and a higher level of depression. In fact, marginal effects analysis shows an association between daily consumption of these beverages and mild depression.

Previous studies have found a significant relation between carbonated beverages and depression, although most of them were focused on adolescents and Asian populations [8,9,36]. However, a recent study from the Brazilian National Survey shows that those with the highest sugar-sweetened beverage consumption had higher depressive symptom scores and those with daily sugar-sweetened beverage consumption showed higher depressive symptoms compared to those with non-daily consumption [11]. Oliván-Blázquez et al. (2021) [12] also concluded that drinking more than one carbonated or sugary drink daily predicts higher depressive symptomatology in individuals with unhealthy behaviors attending Spanish Primary Care settings. Therefore, although our results are not significant, they are close those of previous studies. It is important to point out that WHO recommended reducing the consumption of sugar-sweetened beverages; lower consumption implies a lower intake of “free sugars” and total caloric intake, and therefore, improved nutrition, and less people who are overweight, obese, diabetic or have dental caries [37]. So, it is well studied that carbonated/soft drinks affects both physical and mental health.

In contrast to previous studies, coffee consumption was not associated with depression and anxiety symptoms severity in our sample. The findings of earlier research found that those who consumed coffee weekly or more had significant lower odds of symptoms of depression compared with those who did not consume coffee [14]. Similar results were found in a cross-sectional study, among elderly Asian women [15]. These results are in line with the recommendation of the Food and Drug Administration (FDA), which report that 400 mg/day of caffeine (equal to 4 or 5 cups of coffee) has no negative effects for healthy adults [38]. It is important to pay attention not only to consumption but also the amount of coffee consumed.

In relation to anxiety, previous research found contradictory results. Nouri-Majd et al. (2022) [14] found that those who consumed coffee weekly or more had significantly lower odds of having symptoms. However, in a recent systematic review, caffeine equal to 5 cups of coffee induced anxiety in patients with panic disorder and healthy individuals [39]. Possible reasons for the discrepancy between previous studies and our findings may be that we only collected the units of coffee consumption as a “cup” and not the dose of caffeine (mg/day). Another possible explanation could be the differences in the sample features and the different ways to assess depression and anxiety severity.

No significant associations between tea consumption and depression and anxiety symptom severity were found. This finding is similar to that of a large cross-sectional and prospective study [17] which found that green tea consumption was not linked to the prevalence of depressive symptoms. The multivariable-adjusted odds ratio of depressive symptoms for ≥2 cups/day of green tea was 1.12 compared with <4 cups/week after adjustment for different covariates (trend *p* = 0.67). Nevertheless, many published papers showed the opposite relationship between the consumption of tea and depressive symptomatology. The Singapore Longitudinal Ageing Study (SLAS) found that subjects who consumed three or more cups of tea had a significantly greater reduction in the Geriatric Depression Scale (GDS) symptoms than those who do not drink tea; the odds of worsened GDS depression was significantly lower among those who consumed three or more cups of tea compared to non-tea drinkers, suggesting that tea may have the beneficial effect of preventing the worsening of existing depressive symptoms [40]. Similar results were found by Chen et al., 2022 [41]: habitual tea consumption was associated with minor depressive symptomatology.

Previous results about the relationship between tea consumption and anxiety are divergent and scarce. Similar to our results, Sarris et al., 2019 [42], concluded that L-theanine (an amino acid that can be found in tea leaves) did not demonstrate any beneficial effect over placebo on measures of anxiety in an adult clinical sample with GAD. However, two recent systematic reviews support the opposite findings. Mancini et al., 2017 [19], concluded that green tea influences several psychopathological symptoms, including a reduction in anxiety. Williams et al., 2020 [18], found that 200–400 mg/day L-theanine supplementation has a potential anti-stress effect and anxiety suppressive properties. Most of the studies referenced were carried out in Asian populations, where tea consumption is more extensive compared to Europe.

Our research presents several limitations. First, the sample size is smaller compared to prior studies [11,14,17,33,40]. Second, the specific characteristics of our sample (overweight subjects with subsyndromal depressive symptoms) is not representative of the general population. Third, the percentage of participants who consumed carbonated/soft drinks and tea was low. Four, analysis design does not allow us to draw causal relationships between these beverages and the severity of depressive and anxious symptomatology.

Finally, the dose of caffeine and L-theanine consumption were not assessed. Despite these limitations, our study has several strengths. To the best of our knowledge, this is the first study using longitudinal data examining the association between carbonated/soft drinks, coffee, and tea and depressive and anxiety symptom severity in subjects with subsyndromal depression. In addition, multilevel mixed-effects ordered logistic regression models were proposed, making optimal use of the longitudinal data and the models were adjusted for potential confounding factors. Finally, the subjects were from four different European countries, which may improve the generalizability to other countries.

Our findings provide valuable information about the intake patterns of non-alcoholic beverages and their relationship with anxiety and depressive disorders in European adult populations, specifically, the association between carbonated/soft drinks with sugar and the anxiety severity. This information should be taken into consideration when providing healthy lifestyle recommendations and guidelines to prevent and/or manage this disorder in specific populations.

## 5. Conclusions

The high and regular consumption of carbonated/soft drink with sugar (amount of consumption: ≥1 unit (200 mL)/day) were associated with a higher level of anxiety in a multi-country sample of overweight subjects with subsyndromal depressive symptoms. A trend towards significance was observed in the positive association between both carbonated/soft drinks with sugar and NNSs and depression (amount of consumption: ≥1 unit (200 mL)/day). No relationship was found between coffee and tea consumption and the level of depression and anxiety in this specific population in any of the amounts of consumption analyzed. Further studies may benefit from considering doses (mg/day) of caffeine and L-theanine when exploring the association between coffee and tea with these conditions.

## Figures and Tables

**Table 1:** Sample characteristics at baseline.

Variables	Overall (N = 941)	Range
Demographics characteristics		
Age, in years mean (SD ^1^)	46.8 (12.9)	18–75
Female, *n* (%)	709 (75.3%)	
Site		
Germany, *n* (%)	275 (29.2%)	
Spain, *n* (%)	215 (22.8%)	
The Netherlands, *n* (%)	224 (23.8%)	
United Kingdom, *n* (%)	227 (24.1%)	
Level of education		
Lower education, *n* (%)	91 (9.7%)	
Middle education, *n* (%)	453 (48.1%)	
Higher education, *n* (%)	397 (42.2%)	
Lifestyle variables		
BMI (kg/m^2^) ^2^, mean (SD)	31.3 (3.95)	24–45
MoodFOOD diet score, mean (SD)	51.7 (7.03)	24–71
Alcohol use, mean (SD)	3.88 (3.67)	0–25
Physical activity, mean (SD)	3.61 (2.34)	0–7
Medical comorbidities		
Diabetes, *n* (% yes)	49 (5.2%)	
Clinical characteristics		
History of MDD ^3^ diagnosis, *n* (% yes)	312 (33.2%)	
PHQ-9 ^4^ score, mean (SD)	7.43 (4.23)	0–24
Minimal, *n* (%)	241 (25.6%)	
Mild, *n* (%)	452 (48.0%)	
Moderate, *n* (%)	182 (19.3%)	
Moderate-Severe, *n* (%)	66 (7.0%)	
GAD-7 ^5^ score, mean (SD)	5.76 (4.11)	0–21
Minimal, *n* (%)	430 (45.7%)	
Mild, *n* (%)	356 (37.8%)	
Moderate-Severe	155 (16.5%)	
Group intervention		
Placebo without FB-A ^6^, *n* (%)	234 (24.9%)	
Placebo plus FB-A, *n* (%)	242 (25.7%)	
Supplements without FB-A, *n* (%)	229 (24.3%)	
Supplements plus FB-A, *n* (%)	236 (25.1%)	
Beverages consumption ^7^		
Carbonated/soft drinks with sugar:		
<1/week, *n* (% yes)	731 (77.7%)	
1–6/week, *n* (% yes)	165 (17.5%)	
≥1/day, *n* (% yes)	45 (4.8%)	
Carbonated/soft drinks with NNSs ^8^		
<1/week, *n* (% yes)	602 (64.0%)	
1–6/week, *n* (% yes)	232 (24.7%)	
≥1/day, *n* (% yes)	106 (11.3%)	
Coffee		
<1/day, *n* (% yes)	290 (30.8%)	
1–3/day, *n* (% yes)	534 (56.7%)	
> 3/day, *n* (% yes)	117 (12.4%)	
Tea		
<1/day, *n* (% yes)	627 (66.6%)	
1–3/day, *n* (% yes)	244 (25.9%)	
>3/day, *n* (% yes)	70 (7.4%)	

^1^ Standard deviation; ^2^ body mass index; ^3^ major depressive disorder; ^4^ Patient Health Questionnaire 9 items; ^5^ Generalized Anxiety Disorder 7-Item Scale; ^6^ food-related behavioral activation therapy; ^7^ portion size: carbonated/soft drinks, 200 mL; coffee/tea, a cup; ^8^ non-nutritive sweeteners.

**Table 2:** Associations between beverage consumption and depression and anxiety (at baseline, 6- and 12month follow-up) symptomatology severity categories in multilevel mixed-effects ordered logistic regression adjusted models.

	Depression *			Anxiety *		
Beverage	OR ^1^	*p* Value	CI ^2^ 95%	OR	*p* Value	CI 95%
Carbonated/soft drinks with sugar (200 mL) (ref. < 1/week)						
1–6/week	0.773	0.187	0.527–1.133	0.784	0.280	0.504–1.219
≥1/day	1.965	0.062	0.967–3.993	**2.510**	**0.033**	**1.075–5.864**
Carbonated/soft drinks with NNSs ^3^ (200 mL) (ref. < 1/week)						
1–6/week	1.135	0.411	0.838–1.538	0.947	0.761	0.666–1.347
≥ 1/day	1.570	0.075	0.955–2.581	1.234	0.478	0.690–2.205
Coffee (1 cup) (ref. < 1/day)						
1–3/day	1.070	0.636	0.808–1.417	1.179	0.331	0.846–1.643
>3/day	1.188	0.426	0.777–1.816	1.246	0.393	0.752–2.065
Tea (1 cup) (ref. < 1/day)						
1–3/day	1.044	0.751	0.799–1.364	0.930	0.651	0.679–1.273
>3/day	0.975	0.916	0.613–1.551	1.322	0.309	0.772–2.263

* Model adjusted by time, age, sex, site, educational level, BMI, MooDFOOD diet score, alcohol use, physical activity, diabetes, history of major depressive disorder diagnosis, and group intervention. ^1^ Odds ratio; ^2^ confidence interval; ^3^ non-nutritive sweeteners; Ref: reference. Bold font indicates statistical significance at *p* < 0.05.

## Data Availability

The data presented in this study are available on request from the corresponding author.
